# Transcranial magnetic stimulation over human secondary somatosensory cortex disrupts perception of pain intensity

**DOI:** 10.1016/j.cortex.2012.10.006

**Published:** 2013-09

**Authors:** Patricia L. Lockwood, Gian Domenico Iannetti, Patrick Haggard

**Affiliations:** a Institute of Cognitive Neuroscience, University College London, London, United Kingdom; b Department of Neuroscience, Physiology and Pharmacology, University College London, London, United Kingdom

**Keywords:** Primary somatosensory cortex, Secondary somatosensory cortex, Pain perception, TMS

## Abstract

Pain is a complex sensory experience resulting from the activity of a network of brain regions. However, the functional contribution of individual regions in this network remains poorly understood. We delivered single-pulse transcranial magnetic stimulation (TMS) to the contralateral primary somatosensory cortex (S1), secondary somatosensory cortex (S2) and vertex (control site) 120 msec after selective stimulation of nociceptive afferents using neodymium:yttrium–aluminium–perovskite (Nd:YAP) laser pulses causing painful sensations. Participants were required to judge either the intensity (medium/high) or the spatial location (proximal/distal) of the stimulus in a two-alternative forced choice paradigm. When TMS pulses were delivered over S2, participants' ability to judge pain intensity was disrupted, as compared to S1 and vertex (control) stimulation. Signal-detection analysis demonstrated a loss of sensitivity to stimulation intensity, rather than a shift in perceived pain level or response bias. We did not find any effect of TMS on the ability to localise nociceptive stimuli on the skin. The novel finding that TMS over S2 can disrupt perception of pain intensity suggests a causal role for S2 in encoding of pain intensity.

## 1 Introduction

The ability to quickly and accurately discriminate the intensity and location of a noxious stimulus on the body is essential for survival. Non-invasive functional neuroimaging techniques have shown that noxious stimuli elicit responses in a number of brain structures including primary (S1) and secondary (S2) somatosensory cortices, anterior cingulate cortex (ACC) insular and prefrontal areas (Apkarian et al., 2005). Although some authors consider these regions to be specifically involved in generating painful percepts (e.g., Ploghaus et al., 1999), their functional significance is debated (Mouraux et al., 2011). Although responses in S1 and S2 are thought to subserve the discriminative components of pain sensation (e.g., location and intensity), their functional roles remain largely undefined.

Experimental studies investigating the neural mechanisms of pain intensity discrimination have found evidence for the involvement of both S1 and S2 (Bornhövd et al., 2002; Coghill et al., 1999; Frot et al., 2007; Grundmann et al., 2011; Iannetti et al., 2005; Kanda et al., 2003; Porro et al., 2007; Timmermann et al., 2001; Valmunen et al., 2009). For example, Frot et al. (2007) recorded evoked potentials from intracranial implanted electrodes in S2, and found that S2 responses correlated with perceived pain intensity. Similarly, Bornhövd et al. (2002) reported that BOLD responses in S2 distinguished between different intensities of noxious stimulation. Nevertheless, the role of S2 in pain intensity coding remains controversial. If an area displays a response graded with the stimulus intensity, this does not necessarily imply that the area is important for intensity encoding. The relation could reflect a dimension correlated with perceptual intensity, such as salience or arousal, rather than perceptual intensity itself (e.g., Carmon et al., 1976). For example, almost all the correlations between intensity of pain perception and nociceptive evoked electroencephalography (EEG) responses can be explained as well by accounts based on stimulus salience as by accounts based on pain intensity (Iannetti and Mouraux, 2010). Other studies have also found evidence for S1 involvement in pain intensity encoding (Coghill et al., 1999; Timmermann et al., 2001), but these studies again provide correlational, rather than causal evidence.

More generally, correlations between neural activity and perceptual intensity cannot show that an area or process plays a causal role in intensity encoding. Because transcranial magnetic stimulation (TMS) directly interferes with neural activity in the stimulated area, TMS studies are often thought to offer stronger causal evidence than correlations observed in neuroimaging studies. Table 1 summarises the results of recent relevant studies which stimulated S1 or S2, and assessed effects on judgements of location or intensity of experimental pain. Kanda et al. (2003) reported that TMS over S2 did not affect pain ratings, while TMS over S1 boosted pain ratings. Grundmann et al. (2011) reported that cathodal tDCS delivered to S1 altered sensitivity to cold sensations thought to be mediated by A-delta fibres (Grundmann et al., 2011), but their stimuli were not within the painful range.

To our knowledge, only one previous study has found a significant effect of TMS over S2 on pain intensity. Valmunen et al. (2009) delivered rTMS over a range of cortical sites including S1 and S2. They found that rTMS over S2 but not S1 increased heat pain thresholds on the face. However, Valmunen et al. used thermal contact-heat stimulation, which inevitably involves a combination of both nociceptive and tactile afferent input. Moreover, tactile and nociceptive systems interact strongly at several levels in the CNS. Thus, their findings cannot conclusively demonstrate a selective effect of S2 stimulation on *nociceptive* processing.

Previous research using TMS to investigate the role of S1 and S2 in the perceived location of pain has also yielded mixed findings. Porro et al. stimulated at one of four locations on the hand dorsum, and asked participants to name the stimulated spot (A, B, C or D) on each trial. They found that TMS over S1 significantly impaired participants' ability to localise painful stimuli (Porro et al., 2007). Kanda et al. (2003) used a pointing task in which participants were required to point to the stimulated site on their hand dorsum on an image of their hand. They found no effect of TMS over S1 or S2 on pain localisation judgements (Kanda et al., 2003).

Overall, the existing literature investigating the contributions of S1 and S2 to pain perception is fragmented. To our knowledge no studies have directly compared multiple intervention sites and multiple dimensions of pain perception using an appropriate and fair method that is sensitive to intensity and location encoding. To resolve these ambiguities, we developed an experimental design to systematically investigate the neural basis of sensory pain in the cerebral cortex. Specifically, we sought a design (1) that was causal rather than correlational, (2) that used comparable tasks and psychometric judgements to test two-alternative forced choice judgements of pain intensity and location (3) that would be equally sensitive to contributions of multiple cortical areas and (4) that used nociceptive laser stimulation to specifically activate A-delta fibres without a tactile component. We therefore used single-pulse TMS over S1, over S2, or in a vertex (sham) condition, to disrupt neural processing of pain sensations. Participants judged either the location or the intensity of each stimulus.

## 2 Materials and methods

### 2.1 Participants

Nineteen healthy volunteers (17 right handed, two left handed, 10 females; aged 20–32 years) participated for payment. All participants gave written informed consent, and the local ethics committee approved the experimental procedures.

### 2.2 Stimuli

#### 2.2.1 Thermal stimulation

Painful stimuli were delivered by an infrared neodymium:yttrium–aluminium–perovskite (Nd:YAP) laser with a wavelength of 1.34 m (ElEn, Florence, Italy). This method was used in order to selectively activate A-delta and C nociceptive terminals located in the hairy skin. We used a spot size of 7 mm, a pulse length of 4 msec and two energies (2.75 J and 3.25 J), designed to elicit clear painful pinprick sensations, related to the selective activation of A-delta nociceptors. Previous studies, and a pilot in eight participants, confirmed that this combination of stimulus energy and spot size reliably elicit pinprick sensations. Before the experimental session began, participants reported the intensity of the two stimuli on a numerical scale ranging from 1 to 10, with 1 defined as “no pricking sensation” and 10 as “the most intense pricking sensation imaginable”. The 2.75 J stimulus elicited a mean rating of 3.5 ± 1.0 J, and the 3.25 J stimulus a mean rating of 5.7 ± 1.2 J.

**Fig. 1:**
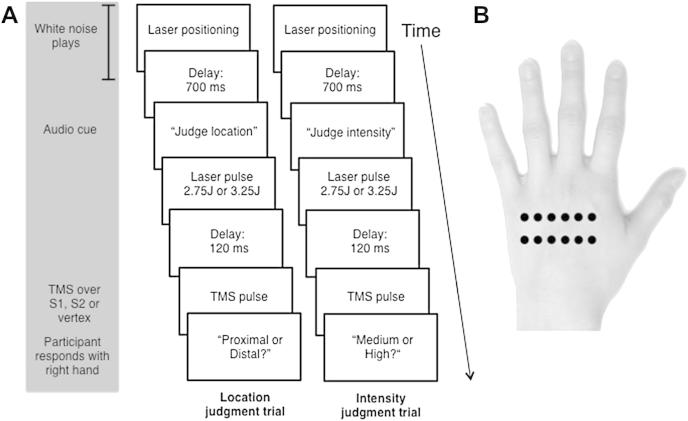
(A) Sequence of events for location and intensity trials. (B) Location of nociceptive stimuli on hand dorsum.

Stimuli were delivered to the left hand dorsum, at either a proximal or a distal locus. The proximal and distal loci were separated by 15 mm with approximately 8 mm between the centres of each site on the proximal or distal line (see Fig. 1). This distance was selected both on the basis of previous studies (Porro et al., 2007; Schlereth et al., 2001) and our pilot study, to elicit an intermediate level of accuracy, avoiding both floor and ceiling effects. After each stimulus participants had to judge whether it was of ‘high’ or ‘medium’ intensity, or whether it was on the ‘proximal’ or ‘distal’ locus (see Experimental procedure for details).

#### 2.2.2 TMS

TMS mapping was conducted in an initial session prior to the main experiment. The motor threshold for each participant was determined by delivering single TMS pulses with a Magstim 200 magnetic stimulator (Magstim, Whitland, Dyfed, UK) using a figure-of-eight coil. The hand motor ‘hotspot’ in the right hemisphere was located by first marking 5 cm lateral and 1 cm posterior to the vertex. The coil was then moved in anterioposterior and mediolateral directions from this location in a 1 × 1 cm grid, delivering single TMS pulses at each site, until motor twitches were obtained in the resting left hand in three out of five successive trials (confirmed by participants' report and experimenter's observation). The mean stimulator output required to elicit motor twitches was 44.8 ± 6.0% of maximum. For the experimental conditions an intensity of 110% of the resting motor threshold was used for all stimulated brain areas (S1, S2 and vertex). The skull vertex was used as a sham stimulation site, to control for the nonspecific effects of TMS such as auditory and sensory artefacts. In sham stimulation, the coil was rotated vertically so that no actual magnetic stimulation was delivered to the brain.

S1 was located by moving the coil posteriorly from M1 until no detectable motor twitches occurred, based on both experimenter observation and reports by the participant. This location was on average 2.4 ± .6 cm posterior to the M1 hotspot. A number of previous studies have localised S1 using this method (Bolognini et al., 2011; Porro et al., 2007). S2 was located as 2.5 cm anterior and 6.5 cm superior to the right preauricular point, again in accordance with previous studies (Bolognini et al., 2011; Kanda et al., 2003).

**Fig. 2:**
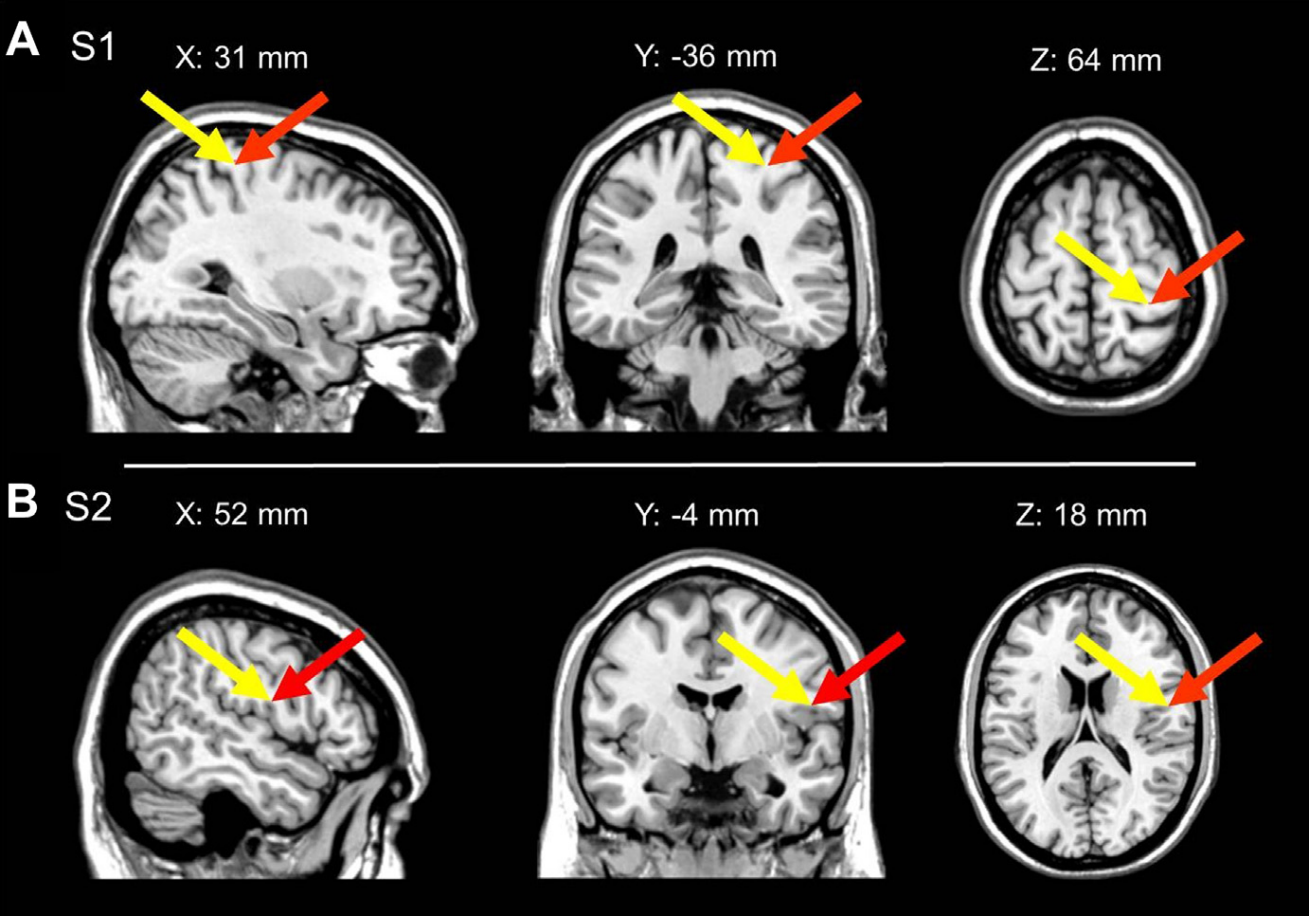
(A) Location of S1 stimulation. (B) Location of S2 stimulation. Red arrows point to putative cortical locations of TMS effects obtained through neuronavigation in nine participants. These locations were calculated by marking the stimulated sites on participants' skull and transforming their locations to Talairach co-ordinates. The trajectory normal to the scalp was followed using the Brainsight neuronavigation system to a depth from the surface equal to that for S1 and S2 responses to nociceptive stimuli in a previous study (yellow arrow = Talairach co-ordinates transposed from left to right hemisphere from Ploner et al. (2009). Note: *in* (A) *X* coordinate from Ploner et al. (2009) has been adjusted by 1 mm to allow registration of display with our stimulation co-ordinates).

In addition, in nine participants these locations were confirmed by using high-resolution structural scans and a neuronavigation system (Brainsight, Magstim, Whitland, Dyfed, UK). We checked in these participants that the stimulated locations corresponded to the Talairach co-ordinates of S1 and S2 previously localised through functional procedures (see Fig. 2). During S1 and S2 stimulation the coil was held 45° to the midline. All participants tolerated the TMS well and there were no adverse effects.

### 2.3 Experimental procedure

To familiarise participants with the experimental procedure and to locate the various brain locations, as well as the appropriate laser intensities and locations, a training session was conducted on a separate day, but within 48 h of the experimental session. Before the training task began, participants were shown a figure of a hand with the hand dorsum sites that would be stimulated during the training and experimental sessions, to ensure that they understood the meanings of the labels ‘proximal’ and ‘distal’.

During this training session, participants completed 20 trials, 10 of the intensity judgement (medium/high) and 10 of the location judgement (proximal/distal), after which feedback was given. If accuracy was below 60%, an additional training block of 10 trials was performed. Once this criterion was reached, the training session was terminated.

During the experimental session, participants' vertex, S1 and S2 were marked with a pen, on the basis of the co-ordinates determined in the training session. The location of S1 was reconfirmed, by delivering one pulse at M1 and one pulse at S1, to ensure that the former produced a detectable motor twitch but that the latter did not. Participants were then seated with their left hand occluded behind a screen. They used a computer mouse held in the right hand to report location/intensity judgements on each trial. At the beginning of the experimental session an example of one medium, one high, one proximal and one distal stimulus were applied to the hand dorsum to remind participants of the stimuli to detect. Participants were instructed to make an un-speeded response by clicking on one of two boxes labelled either ‘medium’ ‘high’ that appeared on screen for the intensity trials, or ‘proximal’ ‘distal’ that appeared for location trials (see Fig. 1 for an example of the sequence of events in an experimental trial). Participants were told that accuracy was important but response time was not. Six sequences of 12 randomised trials, balanced between intensity and location judgements, pulses on the proximal or distal line, as well as laser pulses of medium and high intensity, were created. Intensity and location trials were used in the same blocks to limit any effects of learning, comparison between trials, and expectation. There were never more than three stimuli in succession on either the proximal or distal line, or of medium or high intensity. Each sequence was repeated four times, resulting in 48 trials per block. This method was used to ensure that at least 1 min elapsed between stimulations of the same location, in order to minimise increases of baseline temperature and to limit nociceptor fatigue or sensitization (Iannetti et al., 2004). Block order was randomized among participants. However, one participant received the same sequence for two blocks due to experimenter error.

The spot location for noxious stimulation was controlled by a computer that used two servo-motors to orient the laser beam along two perpendicular axes at the beginning of each trial (see Lee et al., 2009 for details). During laser positioning white noise was played to disguise any potential auditory cues from the servo-motors controlling the laser beam. An audio cue was then played instructing the participant to judge either the intensity or location of the subsequent stimulus, which consisted in a laser pulse of either high or medium intensity. A single TMS pulse was delivered 120 msec after the laser stimulus. This latency was chosen on the basis of the results of previous EEG studies to coincide with the onset of the N1 sensory component of the LEP, which is largely generated in the S1 (Valentini et al., 2012). Each trial lasted a minimum of 5 sec to limit any TMS carry over effects and to ensure that the laser did not stimulate each location more than once a minute (see above). A break of at least 1 min was given at the end of each block in order to change the laser stimulation sequence, reposition the TMS coil and measure the participants' skin temperature. Participants' baseline skin temperature was kept at approximately 30 °C [mean ± standard deviation (*SD*), 30.2 ± .2]. The experimental session consisted of six blocks (one block per each TMS stimulation site repeated twice) of 48 trials, resulting in 288 trials in total. The order of TMS conditions was counterbalanced across participants, and reversed using an ABCCBA design to minimize time-dependent effects.

## 3 Results

### 3.1 Percentage accuracy

One participant spontaneously observed that she had not understood the definitions of the ‘proximal’ and ‘distal’ response categories used in the location judgement task, and was replaced. One further participant showed an outlying pattern of very low accuracy (3.2 *SD*s below the group mean in the vertex control condition, and significantly below chance) on the final block of the experiment (intensity judgement, vertex control). This participant was excluded, but not replaced, leaving a sample of 17 participants.

Preliminary analyses showed that location and intensity judgement tasks had been successfully matched for difficulty (localisation mean % accuracy = 70.3%, *SD* = 8.5; intensity judgement mean % accuracy = 72.3%, *SD* = 6.2).

**Fig. 3:**
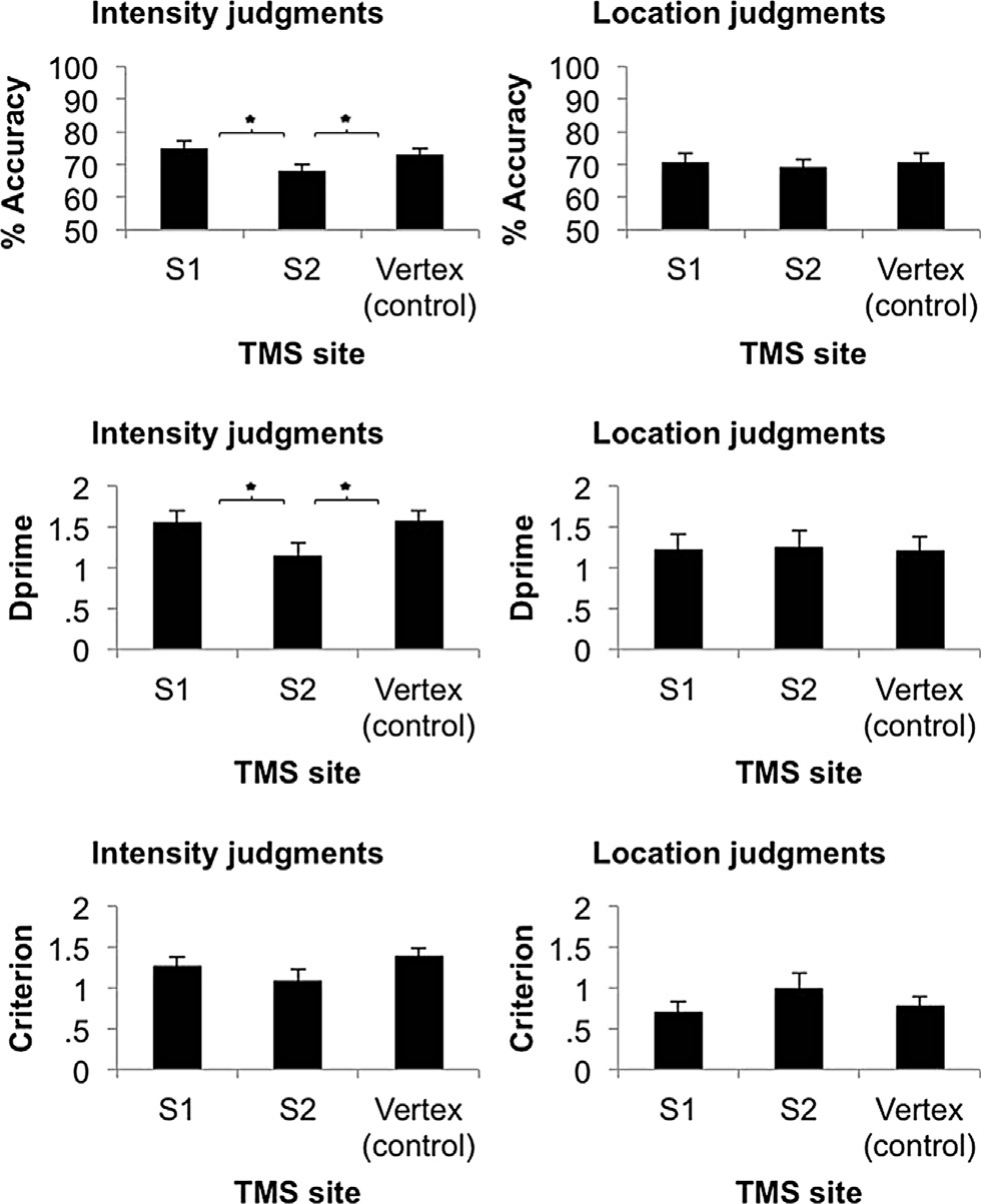
Mean (±S.E.M., *N* = 17) in the intensity judgement condition for percentage accuracy, dprime and response criterion (left panels) and location judgement condition for percentage accuracy, dprime and response criterion (right panels). Asterisks indicate significant differences between conditions using Fisher's LSD test to follow-up overall ANOVA.

Next, we investigated whether areas S1 and S2 contributed to pain perception by simultaneously analysing the accuracy of intensity and location judgements, using one-way multivariate analyses of variance (MANOVA) with a single factor of TMS condition having three levels (S1, S2, and vertex). The MANOVA revealed a multivariate effect of TMS on pain perception which achieved the boundary of statistical significance [Wilks' Lambda = .742, approximated by *F*(4, 62) = 2.50, *p* = .05, Δη^2^ = .139]. Inspection of the canonical structure and standardized canonical coefficients suggested that this effect was largely due to differences across stimulation conditions in intensity judgement, rather than in location judgement (structure: .927, .462; standardised coefficients: 1.229, .519 for intensity and location respectively). Separate follow-up univariate ANOVAs on accuracy of intensity and location judgement, confirmed that this effect was driven by differences in judgements of intensity [*F*(2, 32) = 4.75, *p* = .016, Δη^2^ = .229], not location [*F*(2,32) = .215, *p* = .808, Δη^2^ = .013]. Post-hoc protected comparisons using Fisher's least significant differences test (LSD) were then used to identify significant differences in intensity judgements between TMS conditions. These showed that participants made greater errors in the intensity discrimination task when TMS was applied over S2 (mean 67.8%, *SD* = 9.1) compared to vertex (mean 74.0%, *SD* = 8.1; *p* = .032) and also when TMS was applied over S2 relative to S1 (mean 75.0%, *SD* = 8.9; *p* = .004). In contrast, S1 and vertex TMS conditions did not differ (*p* = .727) (see Fig. 3). Thus, single-pulse TMS over S2 disrupts perception of pain intensity.

#### 3.1.1 Signal-detection analyses

TMS might either alter response sensitivity (i.e., loss of information about whether the stimulus was strong or weak) or response bias (i.e., all stimuli perceived as higher or lower intensity). To distinguish between these possibilities, we also analysed our data using signal-detection theory (Green and Swets, 1966). We arbitrarily defined ‘High’ intensity and ‘Distal’ location as the to-be-detected signals. We computed measures of stimulus sensitivity (dprime) and response bias (criterion) for each participant in each condition. Dprime scores indicate the sensitivity of the participant to the actual intensity or location of the stimulus, while response bias indicates the tendency to respond ‘High’ or ‘Distal’, irrespective of actual intensity/location. The dprime and criterion values for intensity and location judgements were analysed as four dependent variables using MANOVA, as before. The MANOVA again revealed a significant, but now stronger, overall effect of TMS on pain processing [Wilks' Lambda = .530 *F*(8, 58) = 2.71, *p* = .013, Δη^2^ = .272]. The canonical structure (.629, .222, .081, .451 for Intensity dprime, Intensity criterion, Location dprime, Location criterion respectively) suggested that TMS primarily affected sensitivity of intensity perception.

Follow-up univariate ANOVA confirmed that effects of TMS were confined to sensitivity of intensity judgements [*F*(2, 32) = 4.09, *p* = .026, Δη^2^ = .204]. There was no significant effect of TMS site when analysing biases in intensity [*F*(2, 32) = 2.30, *p* = .117, Δη^2^ = .126], sensitivity to location [*F*(2, 32) = .025, *p* = .975, Δη^2^ = .002] nor biases in location [*F*(2, 32) = 2.14, *p* = .134, Δη^2^ = .118]. The significant univariate ANOVA on sensitivity in intensity judgement was followed up using Fisher's LSD. S2 TMS reduced stimulus sensitivity (mean dprime = 1.15, *SD* = .59) relative to vertex control (mean dprime = 1.57; *SD* = .52; *p* = .021) and relative to S1 (mean dprime = 1.56, *SD* = .59; *p* = .011), while S1 stimulation did not differ from the vertex control condition (*p* = .931) (see Fig. 3).

## 4 Discussion

This study is, to our knowledge, the first to use the combination of selective stimulation of nociceptive afferents, balanced psychometric tasks assessing different aspects of pain perception, and single-pulse TMS over multiple cortical areas. We applied single-pulse TMS to cortical areas S1 or S2, or a non-active control site, shortly after laser stimulation. Participants judged the stimulus intensity or location. Our results showed that TMS over S2 disrupted perception of pain intensity, but not of pain location. TMS reduced sensitivity to stimulation intensity, without producing any systematic bias in perceived pain levels. These results are consistent with TMS over S2 disrupting the information-processing that underlies the perception of pain intensity. TMS over S1 had no significant effects on perception of either pain intensity or pain location. We conclude that S2 causally contributes to the ability to discriminate the intensity of a painful stimulus.

Several previous studies had suggested that S2 might code pain intensity (e.g., Bornhövd et al., 2002; Coghill et al., 1999; Frot et al., 2007; Iannetti et al., 2005; Timmermann et al., 2001; Valmunen et al., 2009). Our finding provides clear causal evidence for a role of S2 in the ability to discriminate the intensity of a painful stimulus using nociceptive-selective stimulation and a well-characterised psychometric task. Further, signal-detection analyses showed that TMS over S2 affected judgements of pain intensity by abolishing perceptual sensitivity to stimulus intensity, and not by simply masking pain, or shifting pain levels up or down. Participants' sensitivity to actual stimulus intensity was reduced i.e., the precision of their pain perception. There was no significant bias in pain judgement, either analgesic or hyperalgesic. Our finding confirms previous observations from Valmunen et al. (2009) who reported that rTMS over S2 affected heat pain judgements. Specifically, they found that S2 stimulation both impaired judgements of pain intensity, and reduced perceived pain intensity. We replicated the reduced sensitivity, but not the hypoalgesic bias. Our results also extend their finding, in two ways. First, our result conclusively links S2 to nociceptive processing. Valmunen et al. delivered contact-heat somatosensory stimuli, which inevitably coactivate nociceptive and tactile systems. Given that nociceptive and tactile codes interact at several levels in the nervous system (Melzack and Wall, 1965), the methods used by Valmunen et al. cannot exclude the possibility of indirect effects on pain, as a result of interactions with touch. In contrast, the nociceptive stimulation used in the present study was entirely specific. Second, we show that a single-pulse TMS applied to coincide with the onset of the LEP component is able to disrupt pain coding. Thus, transient disruption of a single stimulus-related cortical process is sufficient to affect pain judgement.

Our experimental design focused primarily on separately comparing S2 TMS to sham vertex TMS, and S1 TMS to sham vertex TMS. Because of the possibility that both S1 and S2 TMS are involved in pain perception, we did not have strong predictions about the differences between S1 and S2 conditions. Interestingly, however, we found that judgements of intensity were significantly disrupted not only when comparing S2 to vertex TMS, but also when comparing S2 to S1 TMS. This result points to distinct roles for S1 and S2 in pain perception, even though they are co-activated in parallel (Liang et al., 2011; Ploner et al., 2009) by nociceptive stimuli. A previous study investigating the role of S1 and S2 in pain intensity discrimination observed that whilst S1 responses were able to gradually encode the intensity of a painful stimulus S2 responses had a more categorical or binary form, showing a sharp increase in amplitude at intensities above the pain threshold (Timmermann et al., 2001). Our results extend these findings by providing evidence that S2 plays a causal role in discrimination of nociceptive stimulus intensity.

Kanda et al. (2003) found that TMS over S1 applied 150 msec and 200 msec post-stimulus increased reports of pain, while TMS over S2 had no effect. However, Kanda et al.'s (2003) task focused on pain detection, rather than coding for graded levels of pain intensity. Indeed, their stimuli remained constant, and they relied on (presumably random) variations in perceived intensity. In the present study we used a two-alternative forced choice pain intensity judgement, which may be more sensitive to the neural encoding of pain levels.

Our TMS did not affect participants' ability to localise noxious stimuli. This result is consistent with the findings of Kanda et al. (2003) but at odds with those of Porro et al. (2007). These last authors observed that TMS over S1 significantly disrupted localisation of painful stimuli. Nevertheless, the role of S1 in pain localisation is still controversial (Apkarian et al., 2005; Bushnell et al., 1999), and several reasons could explain the discrepant results. First, Porro et al. (2007) used mechanical stimuli that activate tactile as well as nociceptive fibres, whilst we used an Nd:YAP laser that selectively activates A-delta fibres but not A-beta fibres. The additional tactile component in Porro et al.'s (2007) study may have contributed to pain localisation, and it may have been this tactile location information that was disrupted by S1 stimulation. Further, we applied single-pulse TMS at 120 msec after a noxious stimulus, based on previous electrophysiological studies of the N1 LEP component (e.g., Valentini et al., 2012), while Porro et al. (2007) applied TMS trains 150 msec and 300 msec after a painful stimulus. They found a significant increase in localisation errors only for the later stimulation. Consequently, S1 coding for location could occur later than S2 coding for intensity. However, previous studies have found evidence for parallel processing of nociceptive stimuli in S1 and S2 (Liang et al., 2011; Ploner et al., 2009), so differences in latency of S1 and S2 coding seem unlikely. Finally, Porro et al.’s location judgements differed from ours in two respects. They used a restricted portion of the hand dorsum between the thumb and index that was not stimulated in our study. Their participants named which of four locations was stimulated, while our participants judged only the proximal/distal dimension of any of 16 stimuli. These differences in stimulation may account for the different results. Additional studies are required to investigate whether S1 and S2 are differentially involved in different types of location judgement and to compare the effects of single-pulse TMS to S1 and S2 applied at various latencies after nociceptive stimulation.

Nevertheless, our study also has limitations. First, the effect observed is relatively small, amounting to a 6.25% decrease in accuracy of intensity judgements following S2 stimulation, relative to vertex control. Pain intensity is notoriously variable, even when nociceptive input remains constant (e.g., Iannetti et al., 2005). Thus, while our results suggest that S2 is involved in the precision or discriminative coding of pain intensity, the clinical importance of this effect remains to be determined. Moreover, clinical interventions generally aim at reducing pain levels, rather than reducing sensitivity to pain. In particular, the absence of any TMS-induced bias in perceived pain level in our data suggests caution about any possible S2 interventions to reduce chronic pain. However, our result does help to answer a classic question in the basic science underlying pain. The question regarding whether parts of the ‘pain matrix’ produce nociceptive sensations and, if so, which ones, has long been controversial. Intracranial microstimulation studies previously suggested that only the insula and opercular regions were involved in the feeling of pain, because these are the only areas which sometimes can evoke pain sensations when stimulated (Ostrowsky et al., 2002). Our results provide direct and causal evidence that S2 is also involved in coding pain intensity.

Second, invasive recording and fMRI studies in humans show nociceptive-related activity both on the S2 surface (e.g., Mazzola et al., 2012), and more deeply in the parietal operculum and insula (e.g., Frot et al., 2007). Given the depth and spatial specificity of TMS effects (Jalinous, 1991) presumably our S2 stimulation mainly affected the superficial area of S2. Our results cannot therefore clarify whether deeper parts of S2, and surrounding operculo-inusular regions also contribute to pain perception. This comment of course applies to other TMS studies of S2, which used similar localisation methods to ours (Bolognini et al., 2011; Kanda et al., 2003). In addition, the effect of TMS depends on the distance between the targeted cortical region and the scalp. This can potentially produce differences in level of effective stimulation at different cortical sites (Stokes et al., 2005). However, we adjusted the stimulus strength according to the motor threshold. If the variation in scalp-cortex distance is mostly variation across individuals, due to factors like overall skull thickness, our approach is sufficient to compensate for this variation. If the variation is due to very local differences in skull and brain anatomy, such that a person may have, for example, a near-surface S2, but a deep S1, our approach could potentially mistake local variations in skull anatomy for functional specialisation. The relevant literature on scalp-cortex distance is quite sparse, and the most systematic study (Stokes et al., 2005) does not specifically report scalp-cortex distances in the areas of S1 and S2. Nevertheless, that study found only minor variations of +2.0 to −1.7 mm in scalp-cortex distance between M1 and parietal sites – the regions closest to S1 and S2 for which data are available. In addition, scalp-cortex distances were strongly correlated across participants between M1 and parietal sites, suggesting that the variability is primarily across individuals at all skull locations, rather than across skull locations within each individual. Therefore, our method of adjusting TMS output according to motor threshold may have partly compensated for this variability.

Finally, we found no evidence for S2 involvement in perception of pain location, and no evidence of S1 involvement in perception of either pain intensity or pain location. These null results should be interpreted with caution. Our results certainly cannot rule out a contribution of S1 to pain perception. Indeed, recent evidence suggests that S1 is the generator of the only EEG feature that is able to predict the subjective pain intensity regardless of stimulus novelty (Zhang et al., 2012).

In conclusion, our findings clarify and extend the results of previous studies correlating S2 activity with perceived pain intensity. In particular, we demonstrate that early-evoked activity in human S2 makes a necessary causal contribution to encoding the intensity of noxious stimuli.

## Figures and Tables

**Table 1:** Summary of previous TMS and tDCS studies that have investigated contributions of S1/S2 to pain intensity and/or location.

	Brain site	TMS/tDCS protocol	Pain stimuli	Response mode	Finding
Kanda et al. (2003)	S1, S2	Double TMS pulses (dTMS), 50 msec apart.	CO_2_ laser stimuli delivered to hand dorsum.	**Location** - point to stimulated site on hand image. **Intensity** - verbally labelled pain intensity on a scale of 0, 1, 2.	**Location** - no significant findings. **Intensity** - dTMS over S1 150–200 msec after CO_2_ laser stimuli increased ratings of pain intensity.
Grundmann et al. (2011)	S1	Anodal, cathodal or sham tDCS at 1 mA current intensity for 15 min.	Cold, warm, thermal pain and mechanical stimuli to skin areas innervated by the radial and median nerve.	**Intensity** - cold, warm, thermal pain and mechanical detection via quantitative sensory testing protocol.	**Intensity** - cathodal tDCS over S1 increased cold detection threshold compared to baseline and sham conditions, and warm detection threshold compared to baseline. No effect for thermal pain or mechanical detection.
Valmunen et al. (2009)	S1, S2	500 rTMS pulses applied at 10 Hz.	Heat pain, cold pain, innocuous warming and cooling on facial skin.	**Intensity** - method of limits and method of levels. Participants indicated the point at which they reached the stimulus intensity threshold with a button press.	**Intensity** - rTMS over S2 increased heat pain thresholds. No effect for S1 TMS.
Porro et al. (2007)	S1	Trains of three TMS pulses, 40 msec apart delivered 150 msec and 300 msec after cutaneous stimulation.	Noxious or non-noxious mechanical stimulation on hand dorsum.	**Location** - identify which of four sites was stimulated.	**Location** - TMS trains 300 msec after stimulation significantly impaired localisation judgements.
